# Access to pediatric medicines in Albania: A qualitative study of family doctors’ perceptions

**DOI:** 10.1371/journal.pgph.0005861

**Published:** 2026-02-10

**Authors:** Eriona Petro, Hendrika A. van den Ham, Aukje K. Mantel-Teeuwisse, Harallamb Martopullo, Iris R. Joosse, Fatima Suleman

**Affiliations:** 1 Local Healthcare Unit, Durres, Albania; 2 WHO Collaborating Centre for Pharmaceutical Policy and Regulation, Division of Pharmacoepidemiology and Clinical Pharmacology, Utrecht Institute for Pharmaceutical Sciences (UIPS), Utrecht University, Utrecht, the Netherlands; 3 Primary Healthcare Center Nr.4, Durres, Albania; 4 WHO Collaborating Centre for Pharmaceutical Policy and Evidence Based Practice, Discipline of Pharmaceutical Sciences, School of Health Sciences, University of KwaZulu-Natal, Durban, South Africa; London School of Economics and Political Science, UNITED KINGDOM OF GREAT BRITAIN AND NORTHERN IRELAND

## Abstract

Access to pediatric medications remains a challenge in Albania, with significant implications for child health outcomes. Family doctors often encounter difficulties in prescribing and ensuring access to appropriate treatments. This study aimed to explore prescriber perspectives on the accessibility of pediatric medicines in Albania and to identify key barriers and facilitators affecting access. Between March and April 2024, semi-structured interviews were conducted with 18 family doctors working in primary healthcare centers in Durrës. Data were analyzed deductively using the Pharmaceutical Value Chain framework, enhancing the methodological rigor of the analysis. Participants identified a range of perceived barriers, including regulatory constraints, pricing issues, limited medicine selection, procurement inefficiencies, and reimbursement challenges. Affordability emerged as a major concern, particularly for low-income families, and directly influenced prescribing behaviors. Shortages, especially of antibiotics frequently necessitated alternative treatments. While some pediatric medicines were available, concerns about the range, formulation, and quality persisted. Facilitators of access included effective patient counseling and clear communication between prescribers and pharmacists. Notably, policy, legislation, and health information technology were not identified as barriers by participants. This study highlights multiple perceived obstacles to pediatric medicine access as reported by prescribers. Findings underscore the need to enforce prescription regulations and update reimbursement policies for pediatric medicines, thereby informing future evidence-based policy interventions aimed at improving child health outcomes in Albania.

## Introduction

Access to healthcare and essential medicines is increasingly recognized as a fundamental human right, critical to strengthening health systems and ensuring positive health outcomes [1]. Achieving equitable access remains a significant challenge in many healthcare settings [2]. Nearly two billion people globally lack access to essential medicines, leading to prolonged illness, avoidable suffering, and preventable deaths [3]. Sustainable Development Goal 3 (SDG3) aims to ensure access to safe, effective, high-quality, and affordable medicines and vaccines for all, highlighting essential medicines for children [4].

Despite this global target, there is a critical shortage of age-appropriate pediatric medicines in low- and middle-income countries (LMICs), where regulatory frameworks may be insufficient [5,6]. Children require specific formulations and dosages to ensure safe and effective treatment, yet most medicines on the market have not been studied or authorized for pediatric use, resulting in the concept of “therapeutic orphans” [7,8]. The WHO Essential Medicines List for Children (EMLc), introduced in 2007, guides the selection of pediatric medicines but is not always implemented consistently across countries, including Albania [9].

Albania, an upper-middle-income country in Southeastern Europe, has a universal public healthcare system in which all residents are assigned to a family doctor at a nearby public health center. While primary care consultations are free of charge, patients frequently incur out-of-pocket payments for diagnostics and, most notably, for medicines. The national health insurance scheme (AMHIF) provides partial or full reimbursement for a limited number of medicines, including coverage for children under 18. However, co-payments are often required, and many pediatric-specific formulations are not included in the reimbursement list. A 2020 WHO report estimated that out-of-pocket health spending accounts for nearly 25% of total health expenditure in Albania, posing a significant burden on low-income families [10–12]. Access to pediatric medicines remains a persistent challenge, as regulatory gaps hinder the availability of age-appropriate formulations compared to EU standards [10]. Recent analyses reveal limited alignment between the AMHIF reimbursement list for children and the WHO Essential Medicines List for Children (EMLc), highlighting gaps in the systematic incorporation of global standards [11]. Weak enforcement of prescription-only regulations further compounds these challenges, as antibiotics, corticosteroids, and other prescription medicines can often be obtained without authorization in community pharmacies, raising concerns regarding both access and patient safety [10]. Although broader issues related to access to essential medicines have been examined in previous research [12–15], there remains a notable gap in understanding the everyday experiences of healthcare providers—particularly family doctors—who must navigate these constraints when prescribing and ensuring access to pediatric treatments.

Family doctors, as primary care providers, are the first point of contact for most pediatric health concerns and are responsible for the majority of prescribing decisions for children. Their role in coordinating care, advising families, and navigating reimbursement pathways positions them uniquely to identify practical barriers that arise when policies do not translate effectively into practice [16,17]. This study therefore aimed to explore the perceptions of Albanian family doctors regarding access to pediatric medicines, with the goal of informing policy development and improving access to pediatric pharmaceuticals in Albania.

## Methods

### Ethics approval and consent to participate

This study involved human participants and Institutional approval was obtained from the Science-Geosciences Ethics Review Board (SG ERB) of Utrecht University (Sci R-23.011) and the Local Healthcare Unit of Durres (Protocol nr.687date 12.12.2023). Participation was voluntary and participants had the option to withdraw from the study at any point.

### Study design

This qualitative, exploratory study involved semi-structured interviews with primary care physicians.

### Sampling of participants

Purposive sampling with a maximum variation strategy was used to select 18 active primary care physicians from healthcare centers in Durrës and Shijak, located within the Durrës district. This district, one of the largest urban areas in Albania, is situated 37 km from the capital, Tirana and is considered representative of the national primary healthcare system. In Shijak, one participant was selected from each of the four public healthcare centers to ensure full coverage, while in Durres, 14 participants were selected from 11 out of 14 centers, prioritizing those serving larger populations and with higher patient volumes. According to recent estimates, the municipality of Durres has a resident population of approximately 153,614, while Shijak has 22,058 inhabitants (see S1 Table). Both the Durrës and Shijak municipalities include rural areas, which were represented in our sample through physicians serving these communities. Participants had specific experience in pediatric care and were identified through the researchers’ professional networks, together with the Durrës Local Healthcare Unit. Recruitment sought to ensure diversity in geographic location and service load by including physicians from urban and rural health centers across the district. This approach provided balanced representation of different practice contexts within the Durrës–Shijak area. Eligible physicians were invited to participate via email and/or text message, and all provided written informed consent before the interviews. Demographic data such as age, gender, and years of experience were collected to describe the sample and enhance contextual understanding of the findings, although not used for subgroup analysis.

### Interview guide

A semi-structured interview guide was developed based on existing literature [17–21], tailored to explore pediatric medication access in Albania. The guide covered topics such as prescribing experiences, challenges in accessing pediatric medicines, medicine substitutions, pricing issues, adherence to clinical guidelines, and the impact of cost on treatment decisions. The interview guide was developed prior to adopting the Pharmaceutical Value Chain (PVC) framework to ensure openness in data capture and avoid constraining participants’ responses within a predefined structure. The PVC framework was instead applied during data analysis to organize emerging themes and provide a systematic interpretive lens. While not used initially to design the interview questions, it offered a coherent structure for synthesizing the results The full interview guide is provided in S1 Text.

### Data collection

Participants were informed of the study’s objectives, provided with an overview of the interview process, and received informed consent documents. Sharing the interview guide in advance aimed to promote transparency and reflection, consistent with qualitative research best practice. This approach enabled participants to articulate more detailed, experience-based perspectives without constraining spontaneity. They were also sent a copy of the interview guide prior to the interview. Interviews were conducted in Albanian by author EP over a two-week period in March–April 2024. Audio recordings were captured using a secure smartphone application. The recordings were transcribed verbatim in Albanian and subsequently translated into English by the interviewer. To ensure accuracy and consistency, a second bilingual researcher (HM) reviewed a random subset of transcripts against the original audio files and translations. Any discrepancies were discussed and resolved through consensus. Both written and verbal consent were obtained before interviews began, which took place face-to-face with only the researcher and participant present. Thematic saturation was identified inductively, as no new concepts emerged in the later interviews, and this was further confirmed during preliminary coding and team discussions.

### Data analysis

Data analysis followed a deductive approach, guided by the Pharmaceutical Value Chain (PVC) framework [17]. The framework’s stages provided a structure for coding and analyzing the data, capturing themes related to access to medicines (see S2 Table). The PVC framework includes ten components, from policy and legislation to health information systems, offering a comprehensive model for analyzing healthcare systems.

Initial coding of five transcripts was performed collaboratively by EP and FS to ensure consistency in code interpretation. The remaining transcripts were independently coded by EP, followed by cross-checking and discussion within the research team to confirm agreement on emerging themes. Final synthesis of the data summarized key themes while maintaining participant confidentiality. Data storage complied with EU General Data Protection Regulation (GDPR).

### Inclusivity in global research

Additional information regarding the ethical, cultural, and scientific considerations specific to inclusivity in global research is included in the S1 Checklist.

## Results

All 18 family doctors approached agreed to participate, with the sample consisting of 13 females and -five males, averaging 9.8 years of professional experience. A detailed overview of participant characteristics is provided in S3 Table. Each interview lasted approximately 30–45 minutes in total, comprising an initial contextual discussion followed by a focused segment lasting 4–16 minutes that addressed specific probing questions on pediatric medicine access. Although participants received the guide in advance, some chose not to respond to every question, which explains the variation in the recorded portion length.

**Fig 1:**
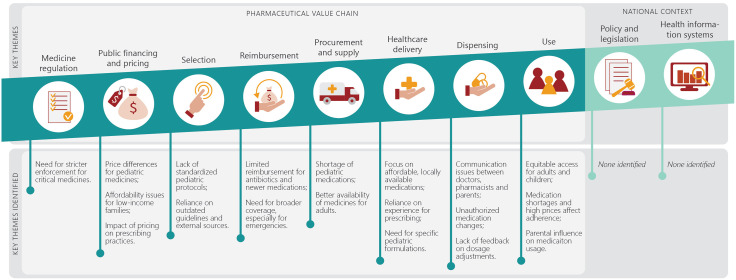
Conceptual Framework of the Pharmaceutical Value Chain (PVC). Diagram illustrating the ten domains of the Pharmaceutical Value Chain framework used to organize qualitative findings on access to pediatric medicines in Albania.

The study identified several sub-themes across eight of the ten components of the PVC framework. No data emerged under the policy and legislation or health information system components, which is consistent with the clinical scope of participants’ roles. This absence highlights the need for future studies involving actors engaged in regulatory and information system functions to provide a more comprehensive analysis of the pharmaceutical value chain. The other eight components and associated sub-themes are discussed below (see Fig 1), with supporting participant quotations. A full collection of quotes is in S4 Table.

### Medicine regulation and enforcement

Participants emphasized that the main concern was not the absence of regulations but their weak enforcement, particularly for medicines commonly used in children, such as antibiotics and corticosteroids. Although these medicines are legally classified as prescription-only under Law No. 105/2014 “On Medicines and Pharmaceutical Service” [22] and Ministerial Order No. 501 of 3 July 2018 [23], participants noted that they are frequently dispensed without prescription, including for children. Stricter enforcement of existing laws was viewed as essential to prevent misuse and antimicrobial resistance in pediatric patients. In contrast, less stringent oversight was considered acceptable for low-risk products such as multivitamins and over-the-counter (OTC) medicines.

- “Personal opinion, we have an uncontrolled access. For me it should be regulated, maybe through the Medicines Agency or some specific law, that any kind of medication cannot be taken. I refer to the cases where the patient goes and takes it [a medicine] without a prescription.” (R4)
- “… which is only uncontrolled access by parents, family members and execution and dispensing by pharmacists. There should have been stricter measures, especially for medications such as antibiotics, cortisones that are given without criteria [clear diagnosis], while for multivitamins and OTC, I don’t believe that a more special rule is needed.” (R4)

### Public financing and pricing

Participants expressed differing views on pricing of medicines for adults and children. Most reported no substantial price differences, whereas others perceived higher prices for pediatric formulations due to their specific dosage forms. Price differences in supplements were also mentioned.

- “No, no, they are at the same values.” (R1)
- “The syrup itself is necessarily more expensive; we can see this very well with levetiracetam.” (R7)
- “Mainly, prices are probably higher in children due to the active ingredient, more specific drug combinations, compared to adults.” (R12)

Some respondents noted that affordability of basic medicines was generally not a problem because of generic options, while others pointed out that low-income families still faced difficulties. Pharmacies were reported to assist parents in identifying lower-cost alternatives through generic substitution.

- “As for the basic medications, no, I have not encountered any difficulties for the parent not to take them due to the issue of cost.” (R6)
- “Mainly with the parents … I make it clear what diagnosis the child has and what he needs. … They are so dedicated that even if they don’t have money, they will borrow just to provide the necessary care.” (R12)
- “There are cases when they [parents] say that we cannot take the entire prescription and we have to make a selection about what is most important and what is not.” (R15)

### Selection

Participants referred to national protocols such as the *Clinical Practice Protocols for Child Growth and Development (0–6 years)* and the *Protocol for the Reimbursable Medicines List*, which were viewed as outdated or insufficient for pediatric care. Several physicians reported using external sources, including PubMed-indexed literature and foreign, especially Italian, guidelines.

- “… the refund list and usage protocol … is from 2019. It is not up to date.” (R10)
- “We simply refer to foreign literature. We access online the sites where the protocols are located.” (R14)
- “… during the period when I worked in pediatrics, I received the Italian protocol and worked based on it.” (R3)

### Reimbursement

Participants discussed several issues related to reimbursement of pediatric medicines. Most agreed that the current list covers many common conditions but lacks breadth, particularly for antibiotics and newer treatments addressing antimicrobial resistance. Challenges were also noted in obtaining reimbursement for emergency medicines.

- “… there should be more, especially as far as antibiotic therapy is concerned; it is very limited.” (R6)
- “I think that taking into account [anti]microbial resistances … there is room for new products in the field.” (R15)
- “Without having a prescription from the doctor … the process of reimbursement of drugs did not work.” (R5)
- “… it needs to be expanded and have more alternatives for each diagnosis so that the doctor has the opportunity to choose and the patient is reimbursed.” (R2)

### Procurement and supply

Views on medicine supply varied. One participant described the national distribution system as effective, whereas most reported shortages and irregular availability of pediatric medicines. Physicians said they often relied on experience to suggest substitutes and, in specific cases, advised parents to obtain medicines from abroad.

- “We have one of the best and most efficient distribution systems … patients don’t have to search in other cities or countries for their medications.” (R9)
- “I think that there are more drugs for adults and it would be good if there were more drugs for children.” (R2)
- “There is currently a shortage of Ventolin (salbutamol) … which is a major problem because you don’t have anything to replace it with.” (R11)

### Healthcare delivery

When prescribing for children, physicians reported considering the illness, availability of medicines, cost, and appropriate pediatric formulations such as liquids or chewable. In economically challenged areas, lower-cost alternatives were often preferred.

- “I prefer to prescribe antibiotics that are available in Albania to make sure they can be found.” (R6)
- “I first check if the medication is available for children. Sometimes there are shortages, like with Vermox (mebendazole).” (R7)
- “Price is a concern, and I try to prescribe medications that patients can afford.” (R1)

Participants also mentioned differing opinions on the efficacy of branded versus generic medicines. Some preferred originator products due to perceived differences in bioavailability, while others were comfortable prescribing generics.

- “They do not always have the same efficiency … bioavailability varies … there are also generic drugs that have a good bioavailability.” (R6)
- “No. In general, working with children is easier because parents are very careful to bring them often for rechecking. Every two or three days they brought them back [for follow-up consultations].” (R3)
- “… a family member from abroad can bring the needed medicine within twenty-four hours.” (R15)

### Dispensing

Participants highlighted the importance of communication between doctors, pharmacists, and parents, particularly during shortages. They reported occasional changes to prescriptions made by pharmacists without consultation, which could cause dosing errors. Lack of feedback from pharmacies was also mentioned.

- “… Helmintox and Vermox have been in short supply … I handled a few cases where a prescription change was made … I provided an alternative.” (R12)
- “I have heard that the medicines were changed in the pharmacy without my knowledge.” (R5)
- “I prefer to be the one who adjusts the dose … very rarely it can happen to be the pharmacist.” (R4)
- “There is no feedback from pharmacies unless I know one personally.” (R10)

### Medicine use

Access to medicines was generally perceived as comparable for adults and children, though adults were thought to have a wider selection due to a broader range of conditions. Participants mentioned that high prices occasionally led parents to omit medicines or skip supportive treatments such as probiotics.

- “There’s no significant difference; it’s pretty much the same. Adults might have more options … but access is similar.” (R14)
- “… if I gave an antibiotic and combined it with a probiotic … there are cases where they did not take the probiotic … the parent justified it was not necessary.” (R9)
- “There are also patients who go to the pharmacy, ask about the price, and do not take it at all.” (R14)

## Discussion

This study examined the perceptions of primary care physicians in Durres, Albania, regarding pediatric medication provision, identifying key barriers related to pricing, selection, availability, reimbursement, and dispensing. A major concern was the lack of regulatory enforcement, particularly for essential medications like antibiotics, which are sometimes dispensed without a prescription. Economic barriers, restrictive reimbursement policies, and outdated pediatric care guidelines were also highlighted. Ineffective communication among healthcare providers and concerns about antimicrobial resistance (AMR) due to a limited list of antibiotics were identified as critical issues requiring urgent attention.

Pricing of pediatric medications was identified as a significant challenge. While physicians generally perceived generic medicines as affordable, this perception should be interpreted within the limits of their clinical experience rather than as an economic evaluation. In daily live many families still struggle with medicine costs, particularly in the absence of comprehensive insurance coverage. National and WHO data consistently show that out-of-pocket spending on medicines in Albania remains among the highest in the region, suggesting that affordability barriers may be underestimated in clinical practice [24]. These economic constraints often affect adherence to treatment and highlight the need for policy reforms to improve access for lower-income families. Lauffenburger et al. (2023) similarly reported that high medication costs reduce parental adherence to long-term treatments, particularly when families face substantial out-of-pocket expenses [25]. Together, these findings underscore the importance of financial protection measures to address the hidden affordability challenges faced by households

Albania’s complex pharmaceutical pricing system reflects broader global challenges, including constraints on public spending, pressures to incentivize pharmaceutical markets, and limitations imposed by intellectual property (IP) frameworks. Similar dynamics have been observed in other post-transition countries, where balancing affordability, access, and international obligations complicates pricing and reimbursement decisions [26,27].

Comparable evidence from neighboring countries in the Western Balkans supports this interpretation. In Serbia, only about half of licensed pediatric medicines were available, and just 51% of WHO Essential Medicines for Children were accessible for children up to 12 years [28]. A regional review by WHO/Europe highlighted that medicines remain one of the main drivers of out-of-pocket payments in Western Balkan health systems. This analysis also showed that for Albania, out-of-pocket payments for outpatient medicines increased from 53% to 76% of total household health spending [24,29]. These findings indicate that the barriers identified in this study reflect broader regional trends, underscoring the need for coordinated regional policies to improve affordability and availability of pediatric medicines.

Reimbursement for pediatric medications, particularly for antibiotics, was also found to be limited, emphasizing the need for expanded coverage and clearer policies, especially in emergency situations. A recent evaluation of Albania’s pediatric medicines in the reimbursement list revealed discrepancies with the WHO essential medicines list for children [30], pointing to the need for regular updates to ensure timely access to essential pediatric treatments.

The study also identified gaps in pediatric care guidelines, with many physicians relying on outdated or external protocols. Bush et al. (2015) emphasized the need for standardized, updated clinical practices [31]. Furthermore, the perceived need for stricter control over antibiotic use suggests that government agencies should enforce more robust regulations and surveillance to track medication dispensing and reduce misuse. Jacobs et al. (2019) found that stricter regulations and public campaigns effectively reduced over-the-counter antibiotic sales [32].

Effective communication between doctors, pharmacists, and parents is vital in pediatric care. However, a persistent lack of coordination—particularly regarding medication availability and dosage adjustments—remains a significant challenge. Research has shown that poor communication across healthcare levels can contribute to medication errors, underscoring the need for improved collaboration to ensure safe and effective treatment for children [33].

The findings align with those from a separate set of interviews with pharmacists, who noted that pediatric medications often cost more than adult medicines, creating financial barriers for families [34]. Similar concerns about medication shortages and substitutions were raised, with pharmacists adopting flexible strategies to ensure continuity of care despite supply disruptions. Improved communication and structured agreements between healthcare providers could further ensure continuity and mitigate these challenges.

While the participants’ responses covered several components of the Pharmaceutical Value Chain (PVC), no responses were given regarding policy and legislation or health information systems. This absence may reflect prescribers’ focus on the practical aspects of their work, rather than higher-level systemic issues. Further research is needed to validate these perceptions and explore additional perspectives, particularly from other stakeholders.

While Albania has its own specific context, the challenges identified—such as limited pediatric formulations, weak regulatory enforcement, and high out-of-pocket spending—are shared by many lower-middle-income and post-transition countries. Similar systems face structural barriers to equitable medicine access, including fragmented financing and insufficient regulation [35,36]. Moreover, despite progress in expanding access to essential medicines, access to newer or specialized treatments remains difficult, even in better-resourced health systems [37]. Thus, the findings from Albania offer relevant insights for policymakers in similar constrained settings.

### Strengths and weaknesses

This study offers valuable insights into pediatric medication access in Albania through qualitative interviews with experienced family doctors. However, not all responses were specifically focused on pediatric care, as many discussions broadened to general healthcare concerns. This may have diluted the focus on children’s healthcare Interview duration varied across participants, which may have influenced the depth of discussion on some pediatric topics. Nevertheless, key themes were consistently identified across interviews, and no substantially new insights emerged in later interviews, suggesting that thematic saturation was reached despite variations in interview length.. The small sample size of 18 physicians from a single district limits the generalizability of the findings. Although the district was purposively selected to reflect diverse urban and rural settings, the results may not be fully representative of the national context and should therefore be interpreted with caution Additionally, while the PVC framework structured the analysis, important areas like policy and legislation were not thoroughly explored. The decision to share interview questions in advance may have led to more reflective responses, rather than spontaneous ones, which should be considered when interpreting the findings. Some responses, such as physicians’ reported use of peer-reviewed journals, may reflect socially desirable answers rather than actual practices.

## Conclusion

This study provides insights into the perceptions of prescribers regarding access to pediatric medications in Durres, Albania. Key challenges identified include medication shortages, pricing issues, and outdated treatment guidelines. While some healthcare providers did not perceive availability or affordability to be significant issues, others highlighted these as major barriers. Updating treatment protocols and standard treatment guidelines, and enhancing communication among healthcare providers are crucial to improving healthcare delivery and child health outcomes. To ensure better access to essential pediatric medications, policy reforms may be needed, especially to update the reimbursement list to include critical medicines for children. Further research is needed to validate these perceptions and explore additional perspectives.

## Supporting information

S1 Text Interview Guide. Semi-structured interview guide used for qualitative interviews with family doctors exploring their perceptions of pediatric medicine availability, prescribing challenges, and adherence.
(DOCX)

S1 Table Resident Population by Municipality. Table showing the total resident population in each surveyed municipality based on data from the Institute of Statistics of Albania (INSTAT).
(DOCX)

S2 Table Pharmaceutical Value Chain Framework (Themes and Sub-Themes). Comprehensive coding framework outlining the major and minor themes derived from interview data, analyzed according to the Pharmaceutical Value Chain (PVC) domains.
(DOCX)

S3 Table Participant Characteristics. Table summarizing demographic and professional characteristics (age, gender, and years of experience) of interviewed healthcare professionals.
(DOCX)

S4 Table Selected Quotes for Identified Barriers and Facilitators. Illustrative quotations from family doctors and pharmacists supporting each theme and sub-theme identified in the qualitative analysis.
(DOCX)

S1 Checklist Inclusivity in Global Research Questionnaire. Completed *PLOS Global Public Health* Inclusivity in Global Research Questionnaire. Additional information regarding the ethical, cultural, and scientific considerations specific to inclusivity in global research is provided in this Supporting Information file.
(DOCX)
