# Supplementary material for: Access to pediatric medicines in Albania: A qualitative study of family doctors’ perceptions
Source: PLOS Glob Public Health. 2026 Feb 10;6(2):e0005861. doi: 10.1371/journal.pgph.0005861 (PMC12890106; doi:10.1371/journal.pgph.0005861)
Supplement: S1 Text — Semi-structured interview guide used for qualitative interviews with family doctors exploring their perceptions of pediatric medicine availability, prescribing challenges, and adherence. (DOCX) [file pgph.0005861.s001.docx]

**S1 Text:** Interview guide

*Introductions*

1. Please tell me a little about yourself.
 • Probe: Years of experience; age; gender

*Experience with Dispensing and Prescribing Medicines for Children*
2. What is your opinion of the medicines available in Albania for use/prescribing for children?
 • Probe: List of medicines on the National Health Insurance List
3. Based on your experience, are children's medicines in the pharmacy mainly patent or generic medicines? What is your perception? Do they have the same effectiveness?
4. Describe your experience of prescribing medications for children.
 • Probe: Difficulties in prescribing (e.g., pricing, registration, formulation issues)
5. If the medication prescribed is not available in the pharmacy and the patient requests a substitution, how do you proceed?
 • Probe: Replacement with another medicine from the same pharmacological class
6. If the prescribed medication is not available in the correct dose, do you communicate with the pharmacist to adjust the dose based on availability?
7. In your opinion, is access to pediatric medicines different compared to adult medicines? Why or why not?

*Pricing-Related Issues*
8. Do you think there is a price difference between medicines for children and those for adults? Why?
9. Do medicine prices affect parents' or caregivers’ ability to access these medicines?
 • Probe: Does pricing influence your prescribing decisions?
10. How does pricing affect how medicines are described in the prescription?

*Adherence to Clinical Guidelines*
11. Please tell us about the sources you use when prescribing medicines for children.
 • Probe: Use of clinical protocols or experience? Which ones? Are they accessible? Are they regularly updated?
12. How would you handle a situation where the medication you prescribed is unavailable, even though it aligns with clinical guidelines?
13. Please share an experience where you faced challenges in adhering to clinical guidelines for pediatric medicines.

*Adherence/Compliance with Medications*
14. What is your experience with children’s adherence to prescribed medications in the context of medicine availability?
15. When a medicine must be replaced due to unavailability, how does this affect adherence?
 • Probe: Positively or negatively? Please provide examples.

Conclusion
16. Is there anything else you would like to share about access to medicines for children?
