# Supplementary material for: Access to pediatric medicines in Albania: A qualitative study of family doctors’ perceptions
Source: PLOS Glob Public Health. 2026 Feb 10;6(2):e0005861. doi: 10.1371/journal.pgph.0005861 (PMC12890106; doi:10.1371/journal.pgph.0005861)
Supplement: S1 Table — Table showing the total resident population in each surveyed municipality based on data from the Institute of Statistics of Albania (INSTAT). (DOCX) [file pgph.0005861.s002.docx]

**S1 Table**. Resident population by municipality.

| Municipality | Population |
| --- | --- |
| Durrës | 153,614 |
| Shijak | 22, 058 |
| Fier | 101,963 |
| Kavajë | 30,012 |
| Lushnjë | 63,135 |
| Tirana | 598,176 |
| Vlorë | 183,436 |

*Source: Institute of Statistics of Albania*. Available from: <https://www.instat.gov.al/en/>. Accessed [5 Dec 2024].
