# Supplementary material for: Access to pediatric medicines in Albania: A qualitative study of family doctors’ perceptions
Source: PLOS Glob Public Health. 2026 Feb 10;6(2):e0005861. doi: 10.1371/journal.pgph.0005861 (PMC12890106; doi:10.1371/journal.pgph.0005861)
Supplement: S2 Table — Comprehensive coding framework outlining the major and minor themes derived from interview data, analyzed according to the Pharmaceutical Value Chain (PVC) domains. (DOCX) [file pgph.0005861.s003.docx]

Pharmaceutical Value Chain Framework (themes and sub themes levels)

**S2 Table** Framework themes and sub themes levels

| **Theme** | **Sub theme-Level 1** | **Sub theme-Level 2** | **Sub theme-Level 3** |
| --- | --- | --- | --- |
| *Medicine regulation* | Regulatory efficiency | Regulation and Accessibility of Medication |  |
|  |  | Regulation and Control of Medication Access |  |
| *Public financing and pricing* | Medicine prices | Variation in Price Between Adult and Pediatric Medicines | Similar Prices |
|  |  |  | Price Differences Between Pediatric and Adult Medications |
|  |  |  | Basic medicines affordable |
|  |  |  | Price variations in supplements |
|  |  |  | Cost Variability Based on Medication Choice |
|  |  | Impact of high price | Impact of high price on accessibility |
|  |  |  | Effect of Pricing on Prescription Adherence |
|  |  |  | Pharmacy Handles Affordability Discussions  Patients socio economic conditions affecting affordability |
|  |  |  | Consultations due to cost and product differences |
|  |  |  | Parents Sacrifice for Children |
| *Selection* | Standard treatment guidelines | Treatment Guidelines: | Accessibility |
|  |  |  | Selective application of updates |
|  |  |  | Need for update |
|  |  |  | Need for Pediatric Protocols in General Practice |
|  |  |  | Adherence to STG dependence on Doctor’s Discretion |
|  |  |  | Challenges in Implementing Guidelines |
|  |  | Impact of Protocol Availability |  |
| *Reimbursement* | Coverage | Range of Reimbursed Pediatric Medicines | Availability of Medications for Specific Chronic Conditions |
|  |  |  | Emergency Room Reimbursement Issues |
|  |  |  | Limitations and Deficiencies in Reimbursement  Need for Expanded Options |
|  | Regulation of insurance schemes | Impact of Reimbursement on Drug Use | Out of pocket expenses |
|  |  | Unclear reimbursement policies |  |
| *Procurement and supply* | Availability of medicines | Equitable availability across the country |  |
|  |  | Limited availability of medicines |  |
|  |  | Greater Availability of Adult Medications |  |
|  |  | Clarification on Generic Medications |  |
|  |  | Perception of generic versus branded drug by health professional | Perceived Efficacy of Generic vs. Off-Brand Pediatric Medications by health professional |
|  |  | Handling unavailable medications | Lack of communication on medicine availability |
|  |  |  | Communication Regarding Medication Availability and Alternatives |
|  |  |  | Selection of Alternative Medications |
|  |  |  | Pharmacists initiated changes |
|  |  |  | Unauthorized Medication Changes by pharmacists |
|  |  |  | Experience-Based Decision Making Adjustments and Solutions to non-available medicines |
|  |  |  | Finding the exact medication when the cases are sensitive |
|  |  | Handling Incorrect Medication Strengths | Communication and Collaboration on Dose Adjustments |
|  |  |  | Lack of Feedback on Medication Dosage Adjustments |
|  |  | Follow-Up and Effectiveness of Medication Replacements |  |
|  |  | Pediatric Drug Shortages |  |
|  |  | Intermittent Supply Issues |  |
|  |  | Addressing Regional Medication Shortages |  |
| Healthcare delivery | Prescribing | Considerations in Prescribing Pediatric Medications | Diagnosis driven prescription |
|  |  |  | Precription based on availability |
|  |  |  | Price & socio-economic factors as Concern in Prescribing Pediatric Medications |
|  |  |  | Formulation and efficacy |
|  |  |  | Cost-Effectiveness Evaluation |
|  |  |  | Consideration of Parental Requests for Foreign Medications |
| Dispensing | Preparing and administering | Doctor's Role in Medication Management |  |
| *Use* | Access to medicines | Variability in access to medicines for adults vs.children | Same access to medicines for adults and children |
|  |  |  | Wider access for adults medicines vs. children medicines |
|  |  | Access issues | Challenges in accessibility |
|  |  |  | Variability in Access to Pediatric |
|  |  |  | Specific Medication Shortages |
|  |  | Cultural influences on medicines use |  |
|  | Adhering and defaulting |  |  |
|  | Rational use of medicines | Communication of healthcare providers in the right execution of the prescription |  |
