# Supplementary material for: Access to pediatric medicines in Albania: A qualitative study of family doctors’ perceptions
Source: PLOS Glob Public Health. 2026 Feb 10;6(2):e0005861. doi: 10.1371/journal.pgph.0005861 (PMC12890106; doi:10.1371/journal.pgph.0005861)
Supplement: S3 Table — Table summarizing demographic and professional characteristics (age, gender, and years of experience) of interviewed healthcare professionals. (DOCX) [file pgph.0005861.s004.docx]

**S3 Table: Participants characteristics**

| Characteristics | Options | n | % |
| --- | --- | --- | --- |
| Age group | 25-34 | 9 | 50% |
|  | 35-44 | 6 | 33.33% |
|  | >45 | 3 | 16.67% |
| Gender | Male | 5 | 27.78% |
|  | Female | 13 | 72.22% |
| Years of experience | 1-9 | 12 | 66.67% |
|  | 10-19 | 3 | 16.67% |
|  | 20-29 | 1 | 5.56% |
|  | >30 | 2 | 11.11% |
