# Supplementary material for: Access to pediatric medicines in Albania: A qualitative study of family doctors’ perceptions
Source: PLOS Glob Public Health. 2026 Feb 10;6(2):e0005861. doi: 10.1371/journal.pgph.0005861 (PMC12890106; doi:10.1371/journal.pgph.0005861)
Supplement: S4 Table — Illustrative quotations from family doctors and pharmacists supporting each theme and sub-theme identified in the qualitative analysis. (DOCX) [file pgph.0005861.s005.docx]

Selected quotes for identified barriers and facilitators

**S4 Table:** Selected family doctors’ quotes

| *Medicine regulation* |
| --- |
| *Regulatory efficiency* |
| *Regulation and Accessibility of Medication* |
| Personal opinion, we have an uncontrolled access. For me it should be regulated, maybe through the Medicines Agency or some specific law, that any kind of medication cannot be taken. I refer to the cases where the patient goes and takes it without a prescription. Simply using drugs and running without a doctor's prescription.-R4 |
| *Regulation and Control of Medication Access* |
| I touch, which is only uncontrolled access by parents, family members and execution and dispensing by pharmacists. There should have been stricter measures, especially for medications such as antibiotics, cortisones that are given without criteria, while for multivitamins and OTC I don't believe that a more special rule is needed.-R4 |
| *Public financing and pricing* |
| *Medicine prices* |
| *Variation in Price Between Adult and Pediatric Medicines* |
| *Similar Prices* |
| No, no, they are at the same values.-R1 |
| So referring to the same active principle No, I have not encountered any difference-R6 |
| There is no difference. Only supplements can make a difference-R8 |
| Price Differences Between Pediatric and Adult Medications |
| I think that for years children have been treated, let's say for antibiotics, but also for some other medications, for other pathologies, mainly with syrup and these are more expensive than when this is in tablet form, which I use for adults. -R2 |
| Then for me I think that pediatric age groups have higher prices, children sell more.-R4 |
| The syrup itself is necessarily more expensive, we can see this very well with Levetiracetams, because there are some children, for example, who cannot take the tablet, so the syrup is many times more expensive.-R7 |
| I haven't noticed to be honest, I think so, I have the impression that I am a bit more expensive compared to drugs for adults.-R9 |
| Price variations in supplements |
| The only thing that is the same as for adults are the supplements, they have price differences based on the company, but they are not necessary, precisely for adults they are their own. -R6 |
| . If we then talk about the supplements that children sometimes need, then there is a bit of a problem, and it would be good if there were cheaper alternatives. -R6 |
| About accessibility, perhaps, they are a bit high, both the supplements and the form of the active principle, that is, the formulation for children that is more expensive.-R7 |
| Impact of high price on accessibility |
| It depends on the economic level. As a center, we cover residents who have an economic standard and it is not a problem for us, but in certain categories we may have. The fact that they are not refunded -R1 |
| Effect of Pricing on Prescription Adherence |
| Yes, it affects, there have been cases -R2 |
| As I said at the beginning, depending on the area we are in, the price also affects-R9 |
| No such cases, but for example, if I gave an antibiotic and combined it with a probiotic, in order to prevent at least the diarrhea that accompanies antibiotics, there are cases where they did not take the probiotic, because they said it was not necessary for treat the disease and at least take the essential medicine, the others manage themselves.-R9 |
| Pharmacy Handles Affordability Discussions  No ~~patient~~ is returned to us. I think that all the discussion about this part is done in the pharmacy-R5 |
| Basic medicines affordable |
| As for the basic medications, no, I have not encountered any difficulties for the parent not to take them due to the issue of cost-R6 |
| *Patients socio economic conditions affecting affordability* |
| It depends on the category of patients. If they are dealing with patients whose parents are unemployed, it is normal that it is unaffordable-R6 |
| *Consultations due to cost and product differences* |
| It has happened. Even when I refer to supplements, it is for this reason, because apart from the cost, the supplements also differ in the amount of vitamins or minerals they have, so they are not equivalent, that is why they have come to be consulted-R6 |
| **Selection** |
| Standard treatment guidelines |
| *Accessibility of Treatment Guidelines:* |
| We don't have protocols, but we have clinical guidelines, which are updated from time to time, but the judgment is always based on anamnesis, clinical signs and not classical guidelines.-R4 |
| Yes, there are such instructions, where you give the dose. Each drug and the data has a clinical use for dosage and for age groups.-R8 |
| Yes, it is an office protocol. I do not know if it is approved by the Ministry of Health.-R9 |
| *Reliance on Online Protocols and external sources* |
| Yes, yes, definitely. It is the main and only source we have at the moment -R1 |
| There is not, in many cases we search online, we search by ourselves.-R2 |
| but I personally, during the period when I worked in pediatrics, I received the Italian protocol and worked based on it.-R3 |
| I have accessed it online myself.-R3 |
| This is because it is my need to update. While the instructions are, we have the instructions. You just wish they were printed. We only have them on the computer and I wish we were more specific.-R4 |
| Normally foreign protocols.Generally, from the books we have received, but there are things you will find yourself, the doctor, to do research or on up to date or on pubmed-R7 |
| Lack of Treatment guidelines: |
| No, we do not have-R1 |
| No we do not have. It would be good to have a guideline for the treatment of pathologies involving the pediatric age.-R2 |
| Then, to my knowledge, we do not have a real protocol, -R3 |
| We don't have a protocol in clinical practice, but I myself study and ask to be updated on the dosages for the protocols for each diagnosis, I certainly follow the protocol.-R5 |
| No, this is the deficiency. We use foreign protocols.-R6 |
| We do not have such a protocol for the pediatric age group.-R8 |
| Need for Pediatric Protocols in General Practice |
| What I think, that every family doctor, despite the fact that we have separate services for adults and children, I think that everyone, even doctors who treat adults, should have access to the understanding of a protocol, that in the moment you come to visit a child, don't refuse it. That you have the opportunity to visit him and you have the opportunity to diagnose him, but it is difficult for you to choose because you do not have the protocol for children in your book, you only have for adults, then if you had such a protocol, in any case you make the visit for children. -R2 |
| For adults, there is a protocol, for children, to be honest, maybe the description sheet for each medication could be like this, but for there to be a protocol, to have the medications only for children, with the doses for children, with the age groups, there is no.-R2 |
| **Impact of Protocol Availability** |
| This is the difficulty of the doctor for adults who refuse to visit children, because if there is a protocol, apply this protocol and it is very simple to visit him, make the diagnosis and give the medication. This prevents me from making pediatric visits, because I do not have a protocol for the medication, because I visit and diagnose him.-R2 |
| Guidelines adherence |
| Selective application of updates |
| Yes, in some specific pathologies yes.-R6 |
| Adherence to STG dependence on Doctor’s Discretion |
| Yes, I personally do it, but it is up to the conscience of the doctor.-R6 |
| Reimbursement |
| *Range of Reimbursed Pediatric Medicines* |
| I think that the medicine found in the list of reimbursable medicines are much more for children than for adults-R2 |
| Then, I think the list of reimbursable drugs, at least in pediatrics, is a little narrower compared to what we have for adults. -R3 |
| In the clinic where I work, medication usually includes OTC drugs, which are medications that are taken without a doctor's prescription, including paracetamol, ibuprofen, anti-influenza, and we rarely get antibiotics through reimbursement. R4 |
| Their range is wide. There are drugs, for use for acute illness and for chronic illness. Drugs for acute diseases are used less, so the list of these drugs is used less. For chronic disease, the list is diverse, as well as for the adult age group-R8 |
| As far as I have seen, a large part of the drugs that are on the list.-R9 |
| For children, taking into account that the primary health system theoretically covers a general part of diseases, without going into the specifics, in practice, the drugs are as they are on the reimbursement list, they are what is needed, in my opinion.-R9 |
| *Limitations and Deficiencies in Reimbursement* |
| Too narrow -R1 |
| I think there should be more medicines for children, especially the reimbursable ones, they were almost non-existent-R5 |
| Yes, there should be more, especially as far as antibiotic therapy is concerned, it is very limited. In other words,-R6 |
| . But there are specific cases which, since they are not often encountered, are not covered by the reimbursement list. -R9 |
| Emergency Room Reimbursement Issues |
| Without having a prescription from the doctor. At least in the emergency when I worked, the work of reimbursable drugs did not work-R5 |
| *Need for Expanded Options* |
| …and I think that it needs to be expanded and have more alternatives for each diagnosis so that the doctor has the opportunity to choose and the patient is reimbursed.-R2 |
| …From what I remember, I don't know how much it might have been updated since then, but I think it could be a bit more extensive as a list of drugs.-R3 |
| But, on the other hand, in addition to the drugs that are in great availability, it is necessary to see or more precisely to see statistically which drug is needed more to have a larger amount of the drug, compared to the rest of the drugs, that maybe we don't need as much as other drugs!  -R9 |
| *Need for Expanded Options of antibiotics* |
| I think about the reimbursement if it is necessary to work more, because there are many families who do not reach anything to receive them, starting many examinations until buying medicines. There are many needy women with major social and economic problems, so the range of reimbursement should be wider, especially for children.-R5 |
| I would like the range of antibiotics that can be given to be added to the list of reimbursable drugs-R6 |
| *Unclear reimbursement policies* |
| Out of pocket expenses |
| I don't know for what reason. But since then there have been prescriptions that have not been reimbursed.-R5 |
| Procurement and supply |
| Availability of medicines |
| Equitable availability across the country |
| Have one of the best and most efficient distribution, because there is no need for the patient to run away and look for it in another city, if not another country, to receive it.-R9 |
|  |
| Clarification on Generic Medications |
| As far as I can see now the difference between patents and generics. Generic is the name of the chemical drug that works?.I'm not sure, to be honest, because I haven't gone into the details of this part.-R9 |
| Perception of generic versus branded drug by health professional |
| Generic. - R1 |
| I think they are more generic- R2 |
| Then I mostly saw that there were patents and even as a doctor myself, in most cases we worked with trade names. -R3 |
| Then, most are mainly patents .-R4 |
| In the market you can find both, that is, patent medicines and generics-R6 |
| Mainly, it seemed to me that they are of the generic type, since they normally have a lower cost. Here I understand that it is only about children, but if I will also speak from experience with adults, I have noticed the progress of Xarelto that was introduced as a patent, even after it came out as a generic, that normally there was a difference.-R7 |
| There are both generic and patent medicines. .-R8 |
| *Limited availability of medicines* |
| I think there should be more medicines for children, especially the reimbursable ones, they were almost non-existent-R5 |
| Normally, it happened to me that I had shortages, especially for antiparasitic drugs, which are not included in the list or even there was a pharmacy shortage,-R7 |
| *Greater Availability of Adult Medications* |
| I think that there are more drugs for adults and it would be good if there were more drugs for children- R2 |
| *Perceived Efficacy of Generic vs. Off-Brand Pediatric Medications by health professional* |
| Almost yes (they have the same efficacy). We have no problems.  -R1 |
| I think they have the same efficiency, I think they should. -R2 |
| There are generic drugs that have the same efficiency in action, but I think they are few. The few I've seen from the use of antibiotics, the ones that haven't been patent, have been like drinking chalk.-R3 |
| and I have not noticed in this decade any difference in efficiency, in patent or generic.-R4 |
| The efficiency is not the same. Those that are patents are better rated and better time tested. And the efficiency is absolutely not the same. Generic drugs have a general effect that is not based on much research. But patents also have studies from which they have reached their use-R5  The greatest efficiency is in patent medicines.-R8 |
| They do not always have the same efficiency, because their bioavailability varies, despite the fact that it may be the same medicine, it is not necessarily the same efficiency. As there are also generic drugs that have a good bioavailability, it is not excluded.-R6 |
| • In efficiency, not exactly, because it is the same medicine, but it was only the price that becomes more affordable.-R7 |
| Handling unavailable medications |
| The case of not finding the drug for long periods of time does not happen. It may happen that it is not an active principle for a certain period, from what pharmacies say due to the lack of warehouses, but it is not that there is no alternative, there is always another alternative.-R6 |
| Lack of communication on medicine availability |
| I personally have not had any cases of them returning to me. But I have heard that the medicines were also changed in the pharmacy without my knowledge-R5 |
| *Communication with pharmacist and parents* |
| Both the parent and the pharmacist, we have communication with both.-R1 |
| It is usually the patient who calls you from the pharmacy and says such and such a medicine, he does not even ask me what I can replace it with. -R3 |
| It is varied, it happens that the pharmacist changes it himself, it happens that the pharmacist takes us on the phone or maybe the parent comes to change the prescription.-R8 |
| *Selection of Alternative Medications* |
| Then we can choose another one, because it is not the case that there is only one medication for a certain pathology. We can select a similar alternative that works for the child.-R2 |
| Yes, normally within the same pharmacological class I have stayed and for this reason I have seen an adequate treatment, it is not that we have had problems in this part  -R7 |
| Yes, but not consulted. It happened, because I see when patients come after the treatment control and they say that I received this product and it is not the same as what I wrote on the prescription.-R9 |
| *Pharmacists initiated changes* |
| Then, maybe sometimes this happens even without the patient coming, without returning to us, because it is the pharmacist who makes the change based on the principle or in cases where no pharmaceutical network is available, we change the type of medication. -R4 |
| In addition to these two cases, there is also a third case that I have to add. There are cases when pharmacies automatically change the preparations themselves.-R9 |
| ***U****nauthorized Medication Changes by pharmacists* |
| I myself do not agree to change the medicine without obtaining the consent of the doctor who gave it. Very rarely a pharmacy may have called me that they do not have the medication and I may have given them something similar to have the same effect. But in most cases they do not notify you.-R5 |
| Yes, there are cases. That's why I wanted to add it to be in the spotlight. He replaces it with another one with almost the same potency as that product, but which he did not consult before. R9 |
| Handling Incorrect Medication Strengths |
| *Communication and Collaboration on Dose Adjustments* |
| No, the doctor, it's generally the doctor. -R1 |
| We also communicate with the pharmacy and have a lot of cooperation with the pharmacy. -R2 |
| There are cases where the pharmacist did it directly, but in general, those who trust the doctor the most, pick you up on the phone and tell you how it should be done. -R3 |
| Then in my daily practice, I prefer to be the one who adjusts the dose and very rarely it can happen me to be the pharmacist who determines it, I try to prescribe it well and if we don't have the same product or the same dose, I talk to the patient about adjusting the dose himself.-R4 |
| but I myself keep this in mind, that if the formula that I have given is different in the dose per milligram per kilogram of weight, with the dose that will be found in the pharmacy, there will certainly be a change in the dosage. -R5 |
| No, it comes back (doctor). At least for my patients, the patient returns to me once.-R6 |
| The point is that it happened to me that even the pharmacy did the dose calculation, but sometimes it happened to me that they made mistakes and took less than it should, and normally the medicine was not efficient enough.-R7 |
| Generally, the pharmacist contacts us again.-R8 |
| *Follow-Up and Effectiveness of Medication Replacements* |
| No, no, the same, that is, the result, the expectation has been good. -R1 |
| No, it's fine, it's fine even if you replace it, because for a certain pathology it's not that there is only one medication, there are several choices, but you try to choose maybe one is cheaper and you think that if this one doesn't exist, is not in the market, then it is replaced by another one.-R2 |
| No. In general, the part of working with children is because parents are very careful to bring them often for rechecking. Every two or three days they brought them back. In the interview others have worked well, that at most you changed a second-generation cephalosporin to a third-generation one and things like that.-R3 |
| It has been followed up and there have been no deteriorations.-R4 |
| I have generally tried to change it with a medication, which would have a similar efficacy, so it has not happened to me.-R6 |
| There was no difficulty, the prognosis went normally.-R8 |
| At the moment when the name is changed, it is not a big deal, so it is a big deal if you took Cefaclor with one company or another, the important thing is that you have Cefaclor. If it works and you are not allergic to that product. On the other hand, when the pharmacy does not have any Cefaclor product, they choose to give a second class to the cefaclor-like antibiotic.R9 |
| In general, the experiences are almost the same, that is, the products that are here, and that are refundable, and the products that they bring from abroad, because they have a relative in Italy or Germany. It is also that placebo effect that patients orientate is better as a product. But the efficiency is approximate, there may be very small differences, maximum one day delay in recovery time. They don't make a big difference to us because we get the result we need with the clinical improvement of the child. But, you parent gives that idea that it got better faster, because it is the most accurate product-R9 |
| Intermittent Supply Issues |
| They are available, but occasionally for specific drugs, there have been shortages. Especially in pharmacies. The idea is that if in the warehouse is missing, it is missing in the pharmacy.-R9 |
| Addressing Regional Medication Shortages |
| • I hope that the specific products, which have certain areas that have certain pathologies. It has been said for a long time that in Durrës parasites are extreme and antiparasitic drugs are often missing, and in my opinion we need to separate the preparations along with the areas, along with the pathologies, so the price should be appropriate as well as the pathology |
| Pediatric Drug Shortages |
| • I believe it is approximate, but there are differences for reasons that perhaps I cannot explain, because often some of the medicines for children are missing.-R9 |
| Healthcare delivery |
| Prescribing  Considerations in Prescribing Pediatric Medications |
| *Diagnosis driven prescription* |
| Personally, when I give a prescription, when I give a drug, I mainly look at the child's diagnosis. The first thing I think is the best medicine he needs for the diagnosis-R5 |
| Okay, for starters it's primary as an idea, it comes down to is it the right class that works for this pathology. -R9 |
| *Prescription based on availability* |
| Personally, I always prescribe the antibiotics found in Albania, so I try to adapt-R6 |
| The first point is: Is this type of medication available for children? For example, it happened to me with medications like Vemox, which was a very big problem because there was a shortage in the market. Also, drugs, for example, against acne, for example. This is not mainly in paediatricians, but there have also been patients who are slightly older.-R7 |
| At the moment it falls directly to a class that interests you in order to act in pathology, then the question comes whether it is available in the pharmacies that are around, because normally patients do not always have the opportunity to run away and they will solve the drugs as close as possible.-R9 |
| *Price & socio-economic factors as Concern in Prescribing Pediatric Medications* |
| Maybe in the price, but so everything that is included, we normally prescribe them.  -R1 |
| Yes, we are thinking about them, we think about them also on the basis of what the patient can afford to buy or afford the cost of the medication.. -R2 |
| Then mainly the prices, because in general most of the drugs are present in the Albanian market, remove any that may be a little more specific, but which are generally not given by the family doctor. But in general there are drugs on the market. The prices are then a bit high for the standard in Albania.-R3 |
| Then, the first thing I think about when I prescribe a prescription is the price because however it can be executed by the patient, the parents and the ease in the patient's pocket. -R4 |
| Then, taking into account the socio-economic conditions, of course he also looked at the price. Although I am not too connected to the price of drugs to know, but in general I am also interested in the economic reason of the patient who is taking it-R5 |
| , the financial part is a problem. In some categories it is still a problem, not in all patients, but in some categories yes, it is a problem-R6 |
| I am self-conscious about this part, I try to give only medicines that have an affordable price, depending on the socio-economic status. That is to say, I look at it more or less out of necessity and secondarily out of the patient's own condition, because this must always be adjusted. . Also for this reason, I give him only what he needs and then give him other advice on how to eat so that he can get the necessary supplements that he could get through drugs that are normally more expensive.-R7 |
| At the moment they are in the pharmacy, you will also take into account the area in which you operate as a doctor, if it is an area with a not very good economic situation, you will normally try to choose a drug that despite the generic name is the same, so Cefixime for Cefixime, but the company changes, the price automatically changes, so something a little cheaper will be chosen to ease the patient's burden. -R9 |
| *Formulation and efficacy* |
| we think since we can choose this and that and they do the same work and have the same effect, we choose ,..-R2 |
| as well as the formulation, of course if the child is vomiting and cannot give a medicine by mouth, so will use the assumption-R5 |
| Find a suitable dosage form for children, I can't say that you can't find it -R6 |
| On the other hand, this can most likely affect the efficiency of the treatment, but it is better to have less efficiency than not to take it at all.-R9 |
| *Cost-Effectiveness Evaluation* |
| In general, we think about cost effectiveness. - R2 |
| The first thing is that, when it is a chronic disease, they take a quality drug. They will rather take the generic drug. In acute illness, we usually prescribe the patent medicine.-R8 |
| *Impact of Drug Prices on Prescription Fulfillment* |
| It is affecting. Usually the problem is that a pediatric prescription goes to 50-60 thousand lek, I'm saying an approximate figure and in general, at least where I had the opportunity to do it, I eliminated some things that he couldn't need , I have given him what he needs. For example, I did not give him food supplements, because they are expensive, taking into account the socio-economic level of the patient, I gave him what was most necessary, so that he at least gets what he needs. As much as supplements, for example ferments, are needed, but still since there was an opportunity.  -R3 |
| Of course, yes.-R5 |
| Dispensing |
| *Doctor's Role in Medication Management* |
| "The doctor must identify what illness the child has. If it’s something minor, parents might think it will improve on its own. But if the diagnosis is more serious, like bronchitis, which could develop into bronchopneumonia, it’s important for the doctor to explain this to the parents. This helps them understand the seriousness of the condition and ensures that the child receives the necessary treatment." (R12) |
| “The case of not finding the drug for long periods of time does not happen. It may happen that it is not an active principle for a certain period, from what pharmacies say due to the lack of warehouses, but it is not that there is no alternative, there is always another alternative.” (R6) |
| - “For example, now there have been shortages in the ant parasitic drugs section, and I didn't know they were in shortage. Not only Helmintox but also Vermox have been in short supply. So I've had a few cases and he's been given a prescription change, for example Albendazole 400mg, I've given him the other alternative.” (R12) |
| “I personally have not had any cases of them returning to me. But I have heard that the medicines were also changed in the pharmacy without my knowledge.” (R5) |
| - “Both the parent and the pharmacist, we have communication with both.” (R1) |
| - “Parents or relatives, pharmacists rarely.” (R10) |
| “Then we can choose another one, because it is not the case that there is only one medication for a certain pathology. We can select a similar alternative that works for the child.” (R2) |
| - “Yes, normally within the same pharmacological class I have stayed and for this reason I have seen an adequate treatment, it is not that we have had problems in this part.” (R7) |
| **Use** |
| Same access to medicines for adults and children |
| I think they are more or less the same. Maybe with reimbursement there are more adult ones, but access to pediatric drugs is about the same, I think. -R3 |
| I think it is the same.-R5 |
| Beyond the list of drugs is the same. In my perception, it is the same.-R6 |
| It is generally the same.-R8 |
| Different access for children and adults |
| In my opinion, there are more drugs for adults than for children -R7 |
| *Access issues* |
| *Variability in Access to Pediatric vs. Adult Medications* |
| *Wider range of adult medications* |
| It is definitely wider, it is wider-R1 |
| *Adhering and defaulting* |
| Cultural influences on medicines use |
| It is the parent's own culture to go to the pharmacy and get the antibiotic-R5 |
| *Impact of non- availability of medicines on adherence* |
| Rational use of medicines |
| Communication Regarding Medication Issues |
| *Parents initiated contact* |
| And the pharmacist, in some cases, we have more direct communication with the patient.-R1 |
| Normally, the patient himself comes and we then give him another, alternative medicine.-R7 |
